# Genome-Wide Identification, Expression Profiling, and Functional Validation of *Oleosin* Gene Family in *Carthamus tinctorius* L.

**DOI:** 10.3389/fpls.2018.01393

**Published:** 2018-10-18

**Authors:** Yubin Lu, Menghan Chi, Lixia Li, Haoyang Li, Muhammad Noman, Ying Yang, Kun Ji, Xinxin Lan, Weidong Qiang, Linna Du, Haiyan Li, Jing Yang

**Affiliations:** College of Life Science, Engineering Research Center of the Chinese Ministry of Education for Bioreactor and Pharmaceutical Development, Jilin Agricultural University, Changchun, China

**Keywords:** oil body, *Ctoleosin*, expression, morphological phenotypes, oil content

## Abstract

*Carthamus tinctorius* L., commonly known as safflower, is an important oilseed crop containing oil bodies. Oil bodies are intracellular organelles in plant cells for storing triacylglycerols (TAGs) and sterol esters. Oleosins are the most important surface proteins of the oil bodies. We predicted and retrieved the sequences of eight putative *C. tinctorius oleosin* (*Ctoleosin*) genes from the genome database of safflower. The bioinformatics analyses revealed the size of their open reading frames ranging from 414 to 675 bp, encoding 137 to 224 aa polypeptides with predicted molecular weights of 14.812 to 22.155 kDa, all containing the typical “proline knot” motif. Reverse transcription quantitative polymerase chain reaction (RT-qPCR) determined the spatiotemporal expression pattern of *Ctoleosin* genes, which gradually increased and peaked during flowering and seed ripening, and decreased thereafter. To validate their role in plant development, we transformed and overexpressed these eight putative *Ctoleosin* genes in *Arabidopsis*. Overexpressing *Ctoleosins* did not affect leaf size, although silique length was altered. *Arabidopsis* transformed with *Ctoleosin*3, 4, and 5 grew longer siliques than did the wild-type plants, without altering seed quantity. The 100-grain weight of the transgenic *Arabidopsis* seeds was slightly more than that of the wild-type seeds. The seed germination rates of the plants overexpressing *Ctoleosin*4 and 6 were slightly lower as compared with that of the wild-type *Arabidopsis*, whereas that in the other transgenic lines were higher than that in the wild-type plants. The overexpression of *Ctoleosin* genes elevated the oil content in the seeds of transgenic *Arabidopsis*. Our findings not only provide an approach for increasing the oil content, but also for elucidating the intricate mechanisms of oil body synthesis.

## Introduction

Oil crop seeds store lipids, primarily triacylglycerols (TAGs), which provide energy for seed germination and seedling growth. The TAGs are contained in specialized organelles called oil bodies (Huang, 1996; Napier et al., 1996; Murphy, 2001), which are present in the seed, pollen, and tapetum of higher plants (Tzen et al., 1993; Huang, 1996). These oil bodies, which have a diameter of 0.2–0.5 μm (Murphy, 2012; Pasaribu et al., 2014), are enclosed in a single layer of phospholipids, unlike most other organelles, which are enclosed in a bilayer membrane (Tzen et al., 1992, 1993; Wu et al., 2010). This single membrane is stabilized by a collection of embedded proteins, three classes of which have been identified, namely oleosin, caleosin, and steroleosin (Lin and Tzen, 2004; Hanano et al., 2006; Tzen, 2012). Among oil body-associated proteins, oleosins, which are the major class, are basic proteins with a molecular weight of about 10–24 kDa (Qu and Huang, 1990). The amino acid sequences of all the oleosins so far studied can be divided into three distinct structural domains: N-terminal amphipathic, central hydrophobic, and C-terminal amphipathic. The hydrophobic domain consists of approximately 70 amino acids and it is highly conserved between species. It is therefore likely to be essential for oleosin for integration into the hydrophobic core of the oil body (Lacey et al., 1998). Both the N- and C-terminal hydrophilic ends of oleosin are much less conserved in their amino acid sequence and located on the surface of the oil body, such that their steric hindrance and electronegative repulsion provide stability to the single phospholipid layer (Huang, 1992; Tzen et al., 1992, 1993; Peng et al., 2003; Purkrtova et al., 2008; Hyun et al., 2013). Oleosins can also modulate oil body size and stability (Huang, 1992; Frandsen et al., 2001; Siloto et al., 2006; Jolivet et al., 2009, 2013; David et al., 2013; Hyun et al., 2013).

To date, a number of cDNA and genomic clones encoding seed oleosins have been isolated from *Zea mays*, *Glycine max*, *Arabidopsis thaliana*, *Sesamum indicum*, *Brassica napus*, *Helianthus annuus*, and other plant species (Keddie et al., 1992; Beaudoin and Napier, 2000; Alexander et al., 2002; Roux et al., 2004; Xu et al., 2004; Chapman et al., 2012). The genes encoding oleosins have been sequenced in many important oilseed crops but they have not yet been assayed in *C. tinctorius* L. Safflower is an oilseed crop of semi-arid regions and occupies a unique position among the oil seed crops due to the high linoleic content of its seed oil (Nikam and Shitole, 1999). Identification of the *Ctoleosin* genes and their functions would be of great significance in breeding. Previously, our team accomplished the *de novo* transcriptome assembly of safflower from which we predicted putative genes for oleosins (Li et al., 2012). Subsequently, we also sequenced the safflower genome, but the data has not yet been released. In this study, eight putative *Ctoleosin* genes retrieved from the safflower genome database were characterized. The expression levels of these *Ctoleosin* genes in different tissues and developmental stages of seeds and flowering were analyzed by RT-qPCR. In addition, the overexpression vector for each gene was constructed and transformed into *Arabidopsis*. The regulatory effect of *Ctoleosin* genes on oil body size and oil content was determined in transgenic *Arabidopsis*.

## Materials and Methods

### Plant Materials

The *C. tinctorius* L. (JiHong No. 1) seeds were purchased from Xinjiang Province of China. They were cultivated in the experimental field of Jilin Agricultural University, Changchun, China. The different tissues of safflower were collected, which included cotyledons, hypocotyls, leaves, stems, flowers, roots, and seeds. The safflower seeds were collected after flowering on day 4 (DAF4), 8 (DAF8), 12 (DAF12), 16 (DAF16), 20 (DAF20), 24 (DAF24), 28 (DAF28), and 32 (DAF32). These samples were immediately frozen in liquid nitrogen and stored at −80°C.

### Sequence Analysis and Prediction of *Ctoleosin* Genes

We predicted and analyzed the sequences of *Ctoleosin* genes from the whole-genome sequence (WGS) database of safflower, which we have accomplished earlier. The physical and chemical properties of the predicted *Ctoleosin* proteins were analyzed by ProtParam online tools ^1^ and the transmembrane domain was predicted by TMHMM2.0^2^. The predicted *Ctoleosin* sequences were checked for the presence of the conserved oleosin domain (PF01277) using Pfam^3^ and SMART^4^. *Arabidopsis* oleosin sequences were collected from The *Arabidopsis* Information Resource (TAIR) 10.0, whereas the other oleosin sequences were obtained from NCBI^5^. The oleosin protein sequences were utilized to identify homologous peptides through BLASTP searches (*e*-value cut-off of 1.0). A phylogenetic tree was constructed by MEGA5.1 using the default parameters (Sarmiento et al., 1997). Analyses of the conserved motifs of *Ctoleosin* sequences were carried out using MEME^6^ with default parameters (Tamura et al., 2011).

### RNA Extraction and cDNA Synthesis

The safflower seeds (0.2 mg) were ground in liquid nitrogen and total RNA was extracted from the various collected tissues and developing seeds using TRIzol (Invitrogen, Carlsbad, CA, United States), according to the manufacturer’s protocols. The RNA quality was determined based on OD_260/280_ values by NanoDrop 2000 (Thermo Fisher Scientific, Beijing, China) and its integrity was detected by 1.2% agarose gel electrophoresis. The total RNA (1 μg) was reverse transcribed into cDNA by the PrimeScript RT Reagent Kit with gDNA eraser (Takara, Japan), following the manufacturer’s protocols, and the cDNA was stored at −20°C.

### Reverse Transcription Quantitative Polymerase Chain Reaction

Reverse transcription quantitative polymerase chain reaction (RT-qPCR) was carried out using the target gene-specific primers (**Table 1**) and SYBR Premix Ex Taq^TM^ kit (Takara, Japan) on Stratagene Mx3000P thermocycler. The six housekeeping reference genes *ACT*, *EF1a*, *GAPDH*, *UBI*, *TUA*, and *TUB* were selected as references for expression analysis in different tissues, and the stability of their expression was evaluated by geNorm and NormFinder software. *Ctoleosin* genes were cloned using a template of cDNA through gene-specific primers. Each reaction was performed in 15 μL reaction mixtures, containing 7.5 μL SYBR Premix Ex Taq, 0.3 μL ROX Reference Dye, 0.3 μL of each gene-specific Primer, 1.5 μL of cDNA, and 5.1 μL ddH_2_O. The PCR profile was set as follows: pre-denaturation at 95°C for 5 min; followed by 40 cycles of 95°C for 20 s and annealing at 62°C for 30 s. The fold-change in relative expression level was calculated using the 2^−ΔΔCT^ method.

**Table 1:** s Primers used for RT-qPCR.

Gene name	Primer sequences
*Ctoleosin1*	Ctoleosin1-F: 5′-ATTGATCGCCGTCTTCATCC-3′
	Ctoleosin1-R: 5′-CCGTCACGTACGAGTAGATCCA-3′
*Ctoleosin2*	Ctoleosin2-F: 5′-ATTTTCAGCCCCGTGTTGG-3′
	Ctoleosin2-R: 5′-CAGAAGAAAACAAACACGGCG-3′
*Ctoleosin3*	Ctoleosin3-F: 5′-TTCAGGAAGAGCCACCAGATCA-3′
	Ctoleosin3-R: 5′-TGAGCCCTCCGTTTTGCAT-3′
*Ctoleosin4*	Ctoleosin4-F: 5′-ATGGACAACGGCCAACTCAA-3′
	Ctoleosin4-R: 5′-CCAGTGGAAACGAAAAAGACGA-3′
*Ctoleosin5*	Ctoleosin5-F: 5′-TTCATCCTCTTCAGCCCCATC-3′
	Ctoleosin5-R: 5′-GCAGTTGACCAGGAACGACAA-3′
*Ctoleosin6*	Ctoleosin6-F: 5′-CAGATACCGTGGACTACGCCA-3′
	Ctoleosin6-R: 5′-CGTACATGCCCATATCGTGG-3′
*Ctoleosin7*	Ctoleosin7-F: 5′-ATCTTCGGCCCTTTGCTGTT-3′
	Ctoleosin7-R: 5′-AACCCATCCCAACGTAGCAAG-3′
*Ctoleosin8*	Ctoleosin8-F: 5′-CCTCATCTTCTTTTCGCCCATC-3′
	Ctoleosin8-R: 5′-ACCCGAAGACACACAGGAATCC-3′

### Construction of Over Expression Vector

*Ctoleosin* cDNA was amplified by PCR from safflower using gene-specific primers (**Table 2**) with *Nco*I and *Hin*dIII restriction sites, and the PCR product was cloned into the respective site of vector pOTB, which was supplied by the Jilin Agricultural University, China. The pOTB-Ctoleosins recombinant plasmids were created, which included the phaseolin promoter, *Ctoleosin* genes, the phaseolin terminator, CaMV35S promoter, bar gene as the selection marker gene, and NOS terminator. The binary vectors pOTB-Ctoleosins were further verified by PCR and *Nco*I/*Hin*dIII restriction analysis, and then transformed into *Agrobacterium tumefaciens* EHA105 competent cells (Hofgen and Willmitzer, 1988; Bailey et al., 2009). The recombinant *Agrobacterium* lines were used to transform *Arabidopsis* plants.

**Table 2:** Primers used for gene clone.

Gene name	Primer sequences
*Ctoleosin1*	Ctoleosin-F1: 5′-CATGCCATGGCTCAAGTCTACCACCACC-3′
	Ctoleosin-R1: 5′-CCCAAGCTTCAAGTCTGGTAGTGCCCG-3′
*Ctoleosin2*	Ctoleosin-F2: 5′-CATGCCATGGCTCACAACCACCACC-3′
	Ctoleosin-R2: 5′-CCCAAGCTTCTAGCGAGTACTATGACCAACTTC-3′
*Ctoleosin3*	Ctoleosin-F3: 5′-CATGCCATGGCGGACTACCACCAC-3′
	Ctoleosin-R3: 5′-CCCAAGCTTAGACTCTAGCAGCCGTATCTT-3′
*Ctoleosin4*	Ctoleosin-F4: 5′-CATGCCATGGACAACGGCCAACTC-3′
	Ctoleosin-R4: 5′-CCCAAGCTTATGTTACACGTCCTGTAAACTCA-3′
*Ctoleosin5*	Ctoleosin-F5: 5′-CATGCCATGGCTGCTGTTACTACTACTCAC-3′
	Ctoleosin-R5: 5′-CCCAAGCTTCTAAGTCCTTCCACCACGTC-3′
*Ctoleosin6*	Ctoleosin-F6: 5′-CATGCCATGGCCACCACATATGACC-3′
	Ctoleosin-R6: 5′-CCCAAGCTTCTAAGTCCGATCTTTTCCACC-3′
*Ctoleosin7*	Ctoleosin-F7: 5′-CATGCCATGGGTACGGTTGAAACGAC-3′
	Ctoleosin-R7: 5′-CCCAAGCTTCTAAGCTCCGACAACCGAC-3′
*Ctoleosin8*	Ctoleosin-F8: 5′-CATGCCATGGCCGATCGGACCAT-3′
	Ctoleosin-R8: 5′-CCCAAGCTTCAAGCACCGGGAGCC-3′

### Generation of Transgenic *Arabidopsis*

*Arabidopsis* seeds were sown in soil, grown for 2 days in the dark and then kept in 16 h photoperiod at 23°C after germination. After 40 days growth, the plants were transformed through the floral dip method and then were harvested for T1 seeds, which was done using 1% basta. The T2 seeds were obtained and bred sequentially until T3 transgenic seeds were harvested.

### Analysis of Morphological Phenotypes

The morphological phenotypes of transgenic *Arabidopsis* were photographed by a digital camera (Nikon, Tokyo, Japan) and an inverted microscope (Olympus IX51, Japan). The phenotypes of leaves, siliques, and seeds of *Arabidopsis* were analyzed from the captured images. The silique lengths, 100-grain weights, and germination rates were measured and statistically analyzed. The above experiments were performed for three biological replicates. Statistical analysis was assessed using the one-way Analysis of Variance (ANOVA), significance level *p* < 0.05^∗^, *p* < 0.01^∗∗^ and *p* < 0.001^∗∗∗^.

### Purification of *Arabidopsis* Oil Bodies

*Arabidopsis* seeds (20 mg) were initially soaked in deionized water (1:5) for 24 h, transferred to 200 μL phosphate-buffered saline (PBS, pH 7.5) and finely ground with a mortar and pestle (Yang et al., 2017). The mixture was filtered, re-dispersed in PBS at pH 7.5, followed by centrifugation at 12,000 × *g* for 20 min to remove the debris. The residual oil bodies were collected, dispersed in 200 μL PBS solution, and centrifuged at 12,000 × *g* and 4°C for 20 min to collect the oil bodies free of extraneous impurities. The recovered pure oil bodies were stored at 4°C.

### Fluorescence Microscopy

The pure oil bodies were diluted using PBS and mixed prior to measurements to ensure their homogeneity. A stock solution of Nile Red (5 mg/L, Sigma, United States) was prepared in absolute ethyl alcohol. The oil body suspensions were stained with an aqueous solution of Nile Red (0.5 mg/L) to visualize neutral lipids and placed in dark for 30 min at 24°C. The stained oil bodies were observed at the magnification of 40× under the fluorescence microscope.

### Measurement of the Oil Body Diameter

The oil bodies were diluted with deionized water to an oil body content of 0.01 weight percentage. The diameter of the oil body was measured by a laser light scattering instrument (PSS NiComp 380ZLS, United States) which ranged from 0.001 to 5 μm. The SD values for oil body suspensions were calculated from every independent experiment. Statistical analysis of the measurements was performed using one-way ANOVA at ^∗^*p* < 0.05, ^∗∗^*p* < 0.01, and ^∗∗∗^*p* < 0.001.

### Lipid Content Detection

For the measurement of lipid content, we added 2 mL of methanol and 4 mL of chloroform to 30 mg of the ground dry seed powder. We mixed the solution for 2 min and treated with ultrasonication for 30 min. The supernatant liquid was transferred to a 20 mL test tube, and 4 mL of trichloromethane solution was added to it. The mixture was centrifuged for 5 min at 12000 × g and the supernatant was collected. Chloroform methanol solution was added to the supernatant and set for 30 min. Finally, the oil layer was collected into a glass vial. The weights of oil and glass vial were determined separately. The formula for measuring oil content was as follows:

$$R=(m_{1}-m_{2})/m\times 100\%$$

where, R is the oil content (%), m_1_ is the total weight of oil and glass vial, m_2_ is the weight of glass vial, and m is the weight of dry seed powders. Statistical analysis was assessed using the one-way ANOVA at ^∗^*p* < 0.05, ^∗∗^*p* < 0.01, and ^∗∗∗^*p* < 0.001.

### Statistical Analysis

These experiments were performed on three biological replicates and the results were visualized using the GraphPad Prism 6.01 software (Inc., La Jolla, CA, United States). Statistical analysis of the measurements was performed using the one-way ANOVA at ^∗^*p* < 0.05, ^∗∗^*p* < 0.01, and ^∗∗∗^*p* < 0.001.

## Results

### *In silico* Analysis of the Identified *Ctoleosin* Gene Family

*Ctoleosin* genes were searched in safflower genome database. They were named as *Ctoleosin*1 to 8 according to their homology. Their predicted molecular weights were 16.8, 14.8, 16.7, 20.1, 21.3, 22.1, 16.0, and 17.0 kDa. Their theoretical isoelectric points were 6.69, 10.11, 9.21, 8.34, 9.17, 9.39, 6.71, and 10.09. The stability index ranged from 23.61 to 42.78 and the aliphatic index from 90.87 to 113.24 (**Table 3**). Most of the Ctoleosins were concentrated in the endoplasmic reticulum, whereas a few *Ctoleosin* proteins were found in the cytoplasm, Golgi apparatus, and plasma membrane. There were two transmembrane regions in Ctoleosin5 and 6, whereas the rest had three transmembrane regions in **Figure 2B**.

**Table 3:** Physical and chemical characteristics of *Ctoleosin* protein.

Protein	Amino acid number	MW (KDa)	Isoelectric point	Aliphatic index	Hydrophilic	Gravg
Ctoleosin1	161	16.8	6.69	90.87	0.080	28.33
Ctoleosin2	137	14.8	10.11	102.55	0.364	29.25
Ctoleosin3	160	16.7	9.21	96.38	0.272	41.63
Ctoleosin4	185	20.1	8.34	124.22	0.780	29.66
Ctoleosin5	211	21.3	9.17	100.24	0.319	23.61
Ctoleosin6	224	22.1	9.39	72.32	−0.103	42.78
Ctoleosin7	148	16.0	6.71	113.24	0.753	22.25
Ctoleosin8	161	17.0	10.09	110.31	0.483	26.24

### Phylogenetic and Structural Domain Analysis of *Ctoleosin*

**FIGURE 1:**
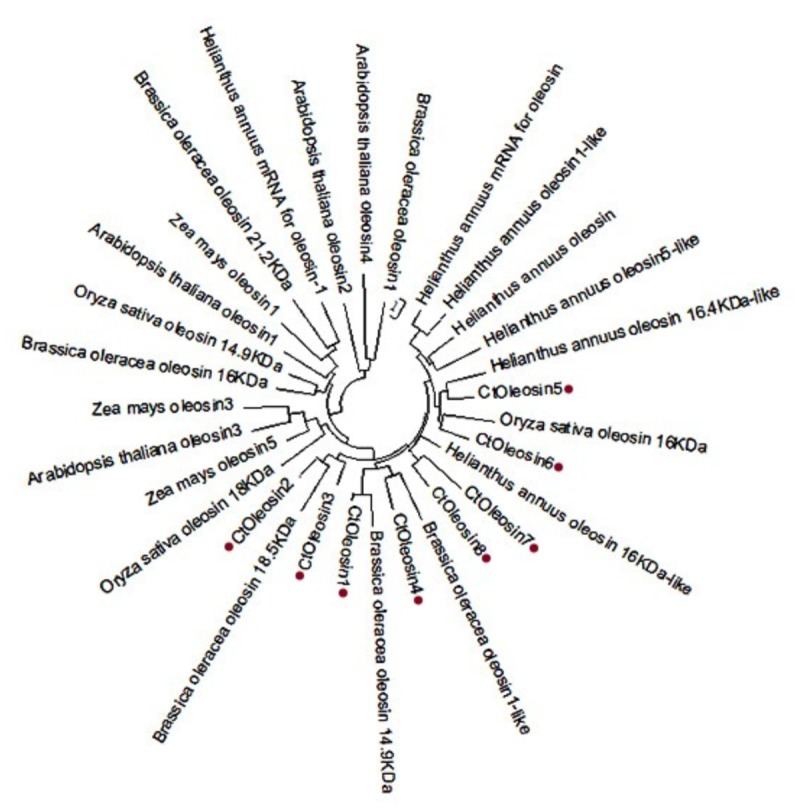
Phylogenetic tree of oleosin genes includes the *Ctoleosin* genes and oleosin genes from *Arabidopsis*, *Helianthus annuus*, *Zea mays* L., *Brassica oleracea* L., and *Oryza sativa.*

**FIGURE 2:**
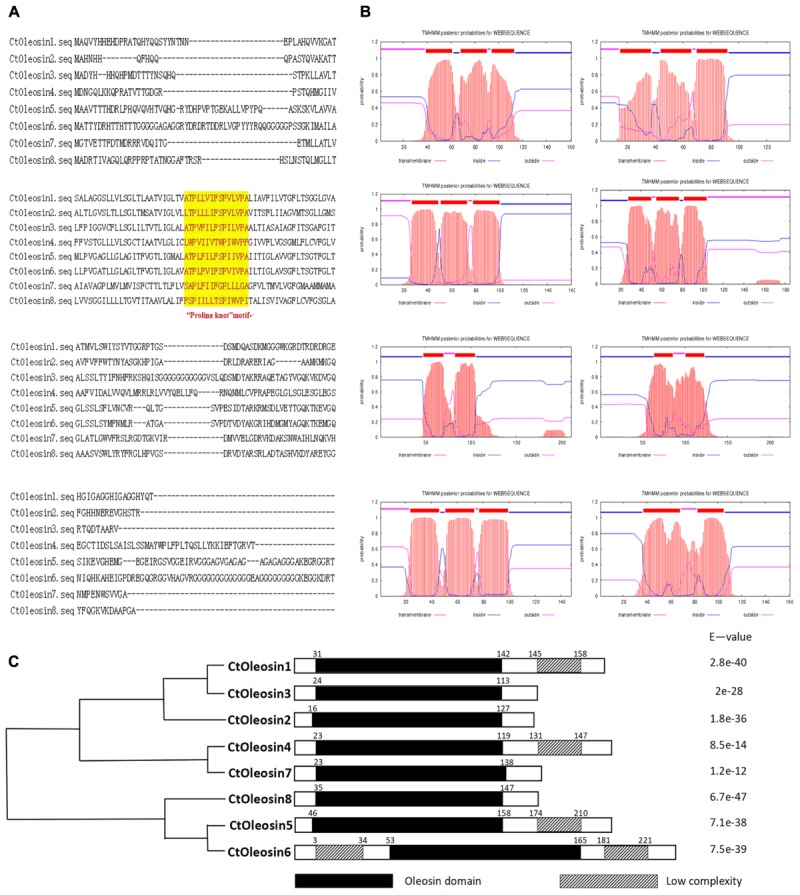
Domain analysis of Ctoleosins. **(A)** Structural domain analysis of the 8 *Ctoleosin* proteins revealed that they all possess the typical proline knot motif (shaded in yellow). **(B)** Transmembrane domain analysis predicted two transmembrane regions in *Ctoleosin5* and 6 while the rest *Ctoleosins* had three regions. **(C)** Conserved oleosin domain (PF01277) analysis of 8 *Ctoleosins* unveiled that all of them are consistent with the oleosin domain.

Using MEGA5.1, we generated a phylogenetic tree from the aligned *Ctoleosin* sequences of *Arabidopsis*, *Helianthus annuus*, *Zea mays* L., *Brassica oleracea* L., and *Oryza sativa* to reveal their evolutionary relationship (**Figure 1**). *Ctoleosin*1 showed higher homology with the 14.9 kDa oleosin of *Brassica oleracea*. *Ctoleosin*2 and 3 were similar to 18.5 kDa oleosin of *Brassica oleracea*. *Ctoleosin*4 was similar to oleosin1-like of *Brassica oleracea*. *Ctoleosin*5 showed more similarity with 16.4 kDa oleosin of *Helianthus annuus*. *Ctoleosin*7 and 8 were similar to 16 kDa oleosin of *Helianthus annuus*. *Ctoleosin* proteins had the same structure as that of the other species; for instance, a hydrophilic N-terminal variable region, a characteristic conserved hydrophobic region, contained a typical “proline knot” motif (PX5SPX3P), and an amphoteric C-terminal variable region (**Figures 2A,C**).

### RT-qPCR Deciphered the Spatiotemporal Expression Pattern of *Ctoleosins* in Safflower Tissues

**FIGURE 3:**
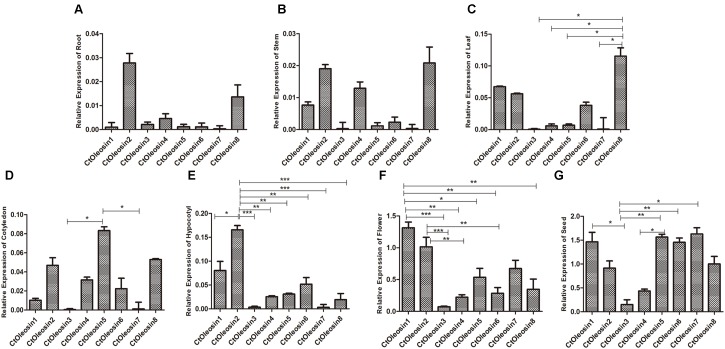
Expression of *Ctoleosin* genes in various tissues. The relative expression levels of *Ctoleosin* genes in root **(A)**, stem **(B)**, leaf **(C)**, cotyledon **(D)**, hypocotyl **(E)**, flower **(F)** and seed **(G)**, which were shown in *Y*-axis, were compared with various tissues. In all graphs, values are average of three biological replicates ± SD, different asterisks indicate significant different applying ANOVA (^∗^*p* < 0.05; ^∗∗^*p* < 0.01; and ^∗∗∗^*p* < 0.001).

RT-qPCR was performed to determine the transcript levels of *Ctoleosin* genes in various tissues of safflower. The most stable reference gene screened by geNorm software was *EF1*α. *Ctoleosin* genes were barely expressed in roots, stems, cotyledons, hypocotyls, and leaves, but their expression level was higher in flowers and seeds (**Figure 3**). The expression level of *Ctoleosin*1 in flowers was 1.29-, 18.7-, 5.95-, 2.45-, 4.68-, 1.95-, and 3.74-fold higher than that of *Ctoleosin*2, 3, 4, 5, 6, 7, and 8, respectively. The expression of *Ctoleosin*1, 5, 6, and 7 was higher than that of *Ctoleosin*2, 3, 4, and 8 on DAF 32.

### *Ctoleosin* Genes Expressed in Different Stages After Flowering of Safflower

**FIGURE 4:**
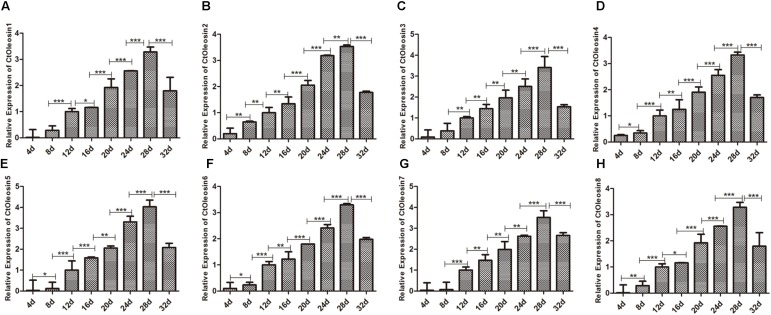
Expression of *Ctoleosin* genes in safflower at different stages after flowering. The relative expression levels of *Ctoleosin1* **(A)**, 2 **(B)**, 3 **(C)**, 4 **(D)**, 5 **(E)**, 6 **(F)**, 7 **(G)**, and 8 **(H)** during different stages after flowering. Values are average of three replicates ± SD. Asterisks indicate significant difference applying ANOVA (^∗^*p* < 0.05; ^∗∗^*p* < 0.01; and ^∗∗∗^*p* < 0.001).

We analyzed the expression pattern of *Ctoleosin* genes in different stages after flowering on days 4, 8, 12, 16, 20, 24, 28, and 32. *Ctoleosin* genes were expressed in all the eight developing stages studied, but their transcript levels were lower at the initial stage of endosperm formation. As the seeds matured, the transcript levels of *Ctoleosin* genes increased progressively and peaked on DAF 28; thereafter, they declined slightly on DAF 32 (**Figure 4**). The expression level of *Ctoleosin*5 was reached 1.08-, 1.13-, 1.17-, 1.21-, 1.22-, 1.14-, and 1.23-fold of that of *Ctoleosin*1, 2, 3, 4, 6, 7, and 8, respectively, on DAF 28.

### *Ctoleosin* Transgene Overexpression in Transgenic *Arabidopsis* Seeds

**FIGURE 5:**
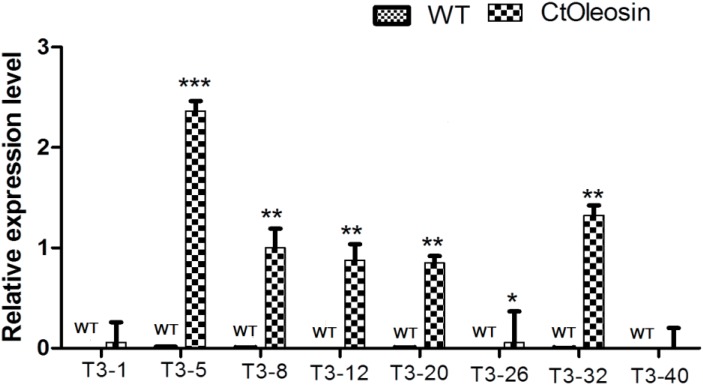
The expression analysis of *Ctoleosin* genes in wild-type *Arabidopsis* seeds and transgenic *Arabidopsis* seeds (Ctoleosin1, 2, 3, 4, 5, 6, 7, and 8). Values are average of three biological replicates ± SD. T3-1: T3 transgenic line for Ctoleosin1; T3-5: T3 transgenic line for Ctoleosin2; T3-8: T3 transgenic line for Ctoleosin3; T3-12: T3 transgenic line for Ctoleosin4; T3-20: T3 transgenic line for Ctoleosin5; T3-26: T3 transgenic line for Ctoleosin6; T3-32: T3 transgenic line for Ctoleosin7; and T3-40: T3 transgenic line for Ctoleosin8. Asterisks indicate significant difference applying ANOVA (^∗^*P* < 0.05; ^∗∗^*P* < 0.01; and ^∗∗∗^*P* < 0.001).

Higher transcript levels of *Ctoleosin* were found in transgenic seeds than that in the seeds of wild-type *Arabidopsis* (**Figure 5**). Transcript level of *Ctoleosin*2 was the highest, followed by that of *Ctoleosin*7, 3, 4, and 5 in transgenic *Arabidopsis* seeds. Expression levels of *Ctoleosin*1 and 6 were the lowest in transgenic *Arabidopsis* seeds. *Ctoleosin* genes were not expressed in wild-type *Arabidopsis* seeds.

### *Ctoleosin* Affected Seed Germination in *Arabidopsis*

**FIGURE 6:**
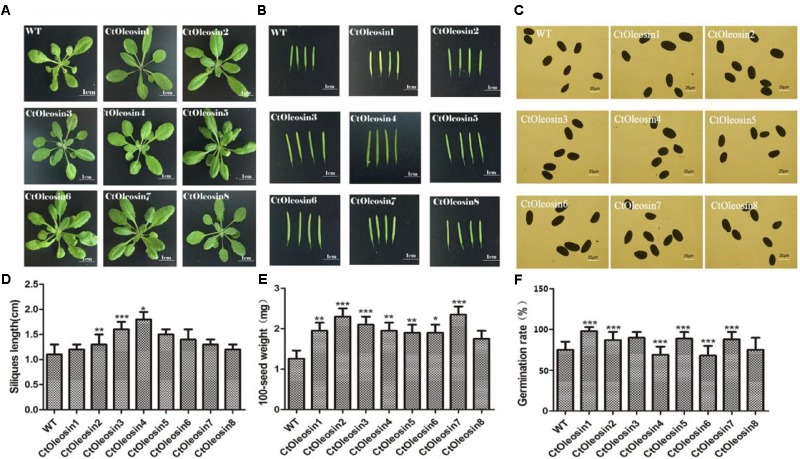
Overexpressing *Ctoleosin* genes in *Arabidopsis* affected tissue development of transgenic plants. **(A)** Leave; **(B)** siliques; **(C)** seeds; **(D)** the siliques length of the transgenic *Arabidopsis*; **(E)** The 100-grain weight of the transgenic seeds; and **(F)** The germination rate of transgenic seeds. Values are average of three biological replicates ± SD. Asterisks indicate significant difference applying ANOVA (^∗^*P* < 0.05; ^∗∗^*P* < 0.01; ^∗∗∗^*P* < 0.001).

The eight *Ctoleosin* genes were overexpressed in *Arabidopsis* under the seed-specific promoter of *Phaseolus vulgaris*. The basta-resistant *Arabidopsis* plants were further validated by PCR with genomic DNA as a template. Fifteen transgenic plants were selected and eight transgenic homozygous lines, whose *Ctoleosin* transcript levels were higher than other seven transgenic plants, were used for morphological observations. As compared with the wild type *Arabidopsis*, leaf size was not significantly altered in the transgenic *Arabidopsis* lines (**Figure 6A**), and silique length increased only in the lines expressing *Ctoleosin*3, 4, and 5 (**Figures 6B,D**). In addition, seed quantity was not significantly altered (**Figure 6C**). The 100-grain weight of transgenic seeds was slightly more than that of the wild-type *Arabidopsis* (**Figure 6E**). The seed germination rates of *Ctoleosin*4- and 6-overexpressing lines were slightly lower than those of the wild-type *Arabidopsis*. In contrast, the germination rates of the lines overexpressing *Ctoleosin*1–3, 5, and 7 were higher than of the wild-type *Arabidopsis*, whereas the germination rate of *Ctoleosin*8 did not differ from that of the wild-type (**Figure 6F**).

### *Ctoleosin* Genes Altered Oil Body Size and Oil Content in *Arabidopsis* Seeds

**FIGURE 7:**
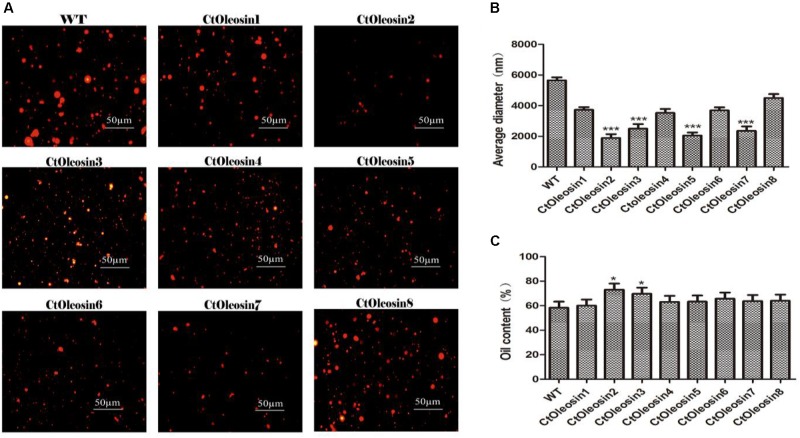
*Ctoleosin* genes altered the oil body size in transgenic *Arabidopsis* seeds. **(A)** The oil body structure in transgenic seeds under the microscope; **(B)** The particle sizes of oil bodies in transgenic seeds. Hydrodynamic diameter expressed by average diameter, size distribution. The average of triplicates was obtained by software. **(C)** The oil content of transgenic seeds. Values are average of three biological replicates ± SD. Asterisks indicate significant difference applying ANOVA (^∗^*p* < 0.05; ^∗∗^*p* < 0.01; ^∗∗∗^*p* < 0.001).

In order to demonstrate that oleosin can regulate the size of the oil body in seeds, we determined the diameter of oil bodies. The oil body diameter was 5.6 μm in wild-type *Arabidopsis*, whereas they were 2.1, 1.9, 4.5, 3.7, 2.5, 3.5, 2.4, and 3.7 μm, respectively, in lines expressing *Ctoleosin*1 through *Ctoleosin*8 (**Figure 7B**). The diameter of the oil bodies in transgenic *Arabidopsis* seeds was less than that of the wild-type plants. The oil bodies were spherical and dispersed uniformly in transgenic seeds (**Figure 7A**). The oil content of transgenic seeds was higher than that of the wild-type seeds. The oil content of lines expressing *Ctoleosin*2 and 3 was higher than that of the other transformants (**Figure 7C**). Accumulation of *Ctoleosin* not only determined the size of the oil bodies in seeds, but also regulated their oil content.

## Discussion

Oilseeds store lipids in oil bodies, which are relatively simple organelles, consisting of a matrix of TAG coated with a phospholipid monolayer embedded with oleosins (Siloto et al., 2006). Oil bodies are formed via an endoplasmic reticulum (ER)-budding process during seed development (Sarmiento et al., 1997; Hsieh and Huang, 2004; Wu et al., 2010) and they are detected as early as the heart stage of embryo development (Siloto et al., 2006; Gallardo et al., 2016). Besides seeds, oil bodies are found in many different tissues and organs (Siloto et al., 2006; Song et al., 2017). Oleosins appear to play an important role in oil seeds, which are the major proteins associated with oil bodies, usually present as two or more isoforms. They have similar structural properties that include a long hydrophobic core organized around a proline knot (Abell et al., 1997). A high degree of similarity is present in but not restricted to the hydrophobic domain and proline knot motif, both of which are essential for the correct targeting of the oil body (van Rooijen and Moloney, 1995; Abell et al., 1997). *Ctoleosins* have a hydrophobic domain and proline knot motif, and this structure can stabilize the oil body. It is presumed that oleosins accumulate throughout seed development (Siloto et al., 2006; Song et al., 2017), and that they stabilize the oil body by steric hindrance and electronegative repulsion (Tzen et al., 1993; Wu et al., 2010). Moreover, they prevent oil body coalescence during the process of seed maturation and affect the final size of oil bodies (Cummins et al., 1993; Leprince and Hoekstra, 1998; Schmidt and Herman, 2008). Siloto et al. have clearly demonstrated that oleosin accumulation regulates the size of oil body by studying the effect of reduced oleosin accumulation on seed germination and TAG accumulation (Siloto et al., 2006). Oleosin silencing in *Arabidopsis* resulted in the formation of enlarged oil bodies as compared with that of the wild-type plants (He and Wu, 2009). It has been suggested that the size of the oil bodies is controlled by the relative contents of the oleosin (Ting et al., 1996; Shimada et al., 2008; Wu et al., 2010).

In previous studies, when the major oleosin was suppressed in *Arabidopsis* seeds, oil bodies were found to be larger and TAG accumulation levels were reduced (Siloto et al., 2006; Shimada et al., 2008). Several lines of evidence support this statement, including the variability of oleosins and oil body size in maize lines with different oil content (He and Wu, 2009; Wu et al., 2010). In the present study, we verified that *Ctoleosin* genes decrease oil body sizes and alter oil content by overexpressing the genes of the *Ctoleosin* family in transgenic *Arabidopsis* seeds. These results indicate that *Ctoleosin* genes play an important role in altering oil body size and oil content. Simultaneously, the introduction of exogenous oleosin in the transgenic lines indicates that it can replace the modified oleosin on the surface of oil body. Replacing natural oleosins by recombinant modified oleosin can provide novel insights into the targeting mechanisms, TAG sequestration in oil bodies, and *in vivo* features of the oil body surface (Siloto et al., 2006). This is certainly useful in many studies on oleosin function and molecular interactions. The use of modified oleosin as oil body platforms for the production of recombinant exogenous proteins has wide prospects of application.

## Author Contributions

JY and HaiL conceived and designed the experiments. YL and MC conducted most of the experiments. KJ, YY, MN, HaoL, WQ, XL, and LD participated in experiments and data collection. All authors read and approved the final manuscript.

## Conflict of Interest Statement

The authors declare that the research was conducted in the absence of any commercial or financial relationships that could be construed as a potential conflict of interest.

## Footnotes

1 http://www.expasy.org/

2 http://www.cbs.dtu.dk/Services/TMHMM

3 http://pfam.xfam.org/

4 http://smart.embl-Heidelberg.de/

5 https://www.ncbi.nlm.nih.gov/

6 http://meme-suite.org/tools/meme
